# Supplementary material for: A data-driven generative strategy to avoid reward hacking in multi-objective molecular design
Source: Nat Commun. 2025 Mar 11;16:2409. doi: 10.1038/s41467-025-57582-3 (PMC11897179; doi:10.1038/s41467-025-57582-3)
Supplement: Supplementary file 1 — Supplementary Information [file 41467_2025_57582_MOESM1_ESM.pdf]

Supplementary Information

A Data-Driven Generative Strategy to Avoid Reward Hacking in Multi-objective Molecular Design

Tatsuya Yoshizawa[1,2], Shoichi Ishida[1], Tomohiro Sato[2], Masateru Ohta[3], Teruki Honma[2], Kei Terayama[1,4,5*]

1. Graduate School of Medical Life Science, Yokohama City University, 1-7-29, Suehiro-cho, Tsurumi-ku, Yokohama, 230-0045, Kanagawa, Japan.
2. RIKEN Center for Biosystems Dynamics Research, 1-7-22, Suehiro-cho, Tsurumi-ku, Yokohama, 230-0045, Kanagawa, Japan.
3. HPC- and AI-driven Drug Development Platform Division, RIKEN Center for Computational Science, 1-7-22, Suehiro-cho, Tsurumi-ku, Yokohama, 230-0045, Kanagawa, Japan.
4. RIKEN Center for Advanced Intelligence Project, 1-4-1, Nihonbashi, Chuo-ku, 103-0027, Tokyo, Japan.
5. MDX Research Center for Element Strategy, Tokyo Institute of Technology, 4259, Nagatsuta-cho, Midori-ku, Yokohama, 226-8501, Kanagawa, Japan.

*Corresponding author(s). E-mail(s): terayama@yokohama-cu.ac.jp;

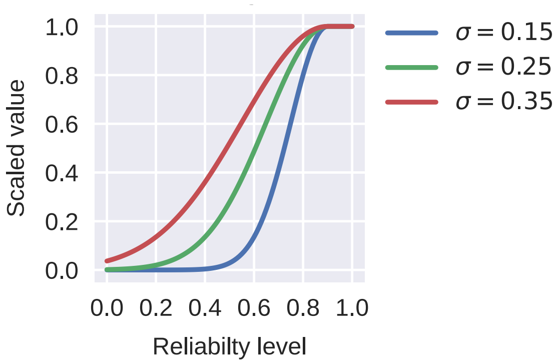

Figure S1. Scaling function to standardize reliability levels. Reliability levels are scaled in calculating the DSS (Degree of Simultaneous Satisfaction of prediction reliability and multiple property optimizations) score. As the scaler, MaxGaussian(μ, σ) was used referring to previous study[1]. This function returns 1 for values greater than μ and approaches zero according to a Gaussian function for values less than μ. In this study, the value of μ is set to the upper limit of search space of reliability levels. For σ, three patterns were prepared to account for prioritization among properties in adjusting the reliability levels. Reducing the σ makes this scaler stricter and more sensitive to response to fluctuations in reliability levels. The shown figure represents the scaling functions when μ is set to 0.9 and σ is set to three patterns, 0.15, 0.25, and 0.35.

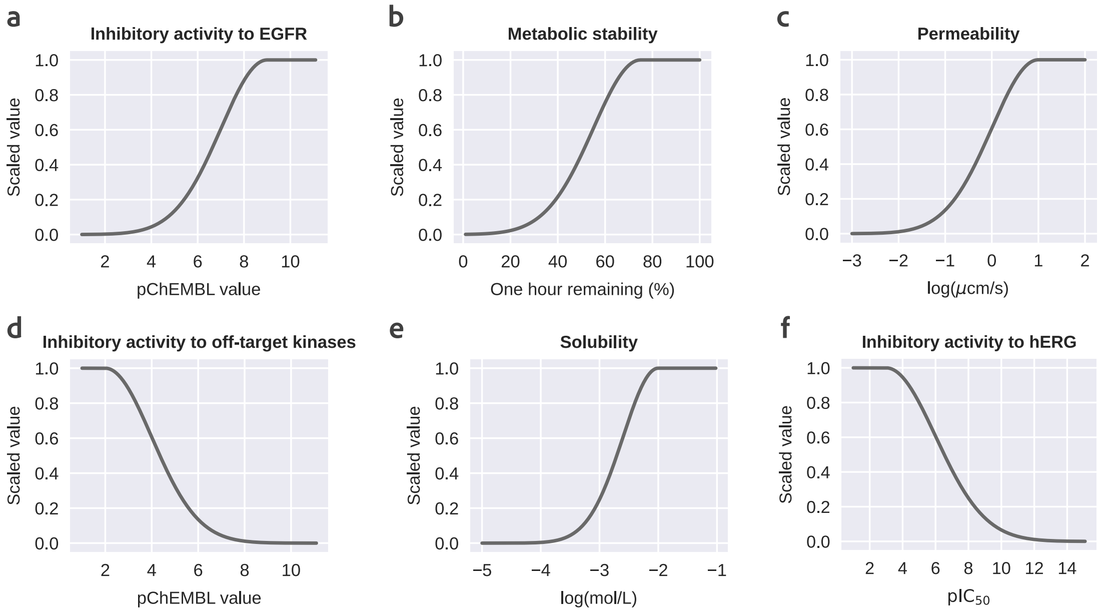

Figure S2. Scaling functions for properties. a-f represent functions for inhibitory activity against epidermal growth factor receptor (EGFR), metabolic stability, permeability, inhibitory activity against off-target tyrosine kinases, solubility, and inhibitory activity against human ether-a-go-go-related gene channel (hERG), respectively. Two types of scaling functions, MaxGaussian(μ, σ), and MinGaussian(μ, σ), were used with reference to previous study[1].

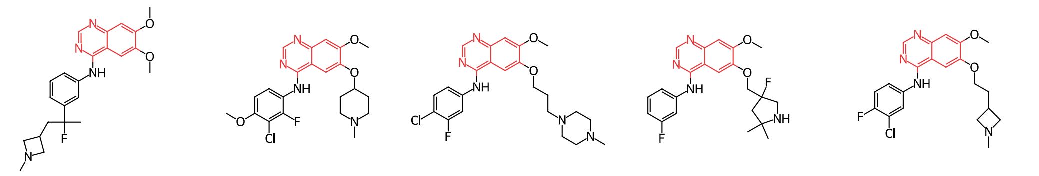

Pred.	7.96	71.3	7.96	8.12	69.1	5.83	8.04	70.5	5.70	7.92	73.8	5.92	8.02	72.6	4.71
Rel.	0.66	0.53	0.53	0.79	0.60	0.60	0.80	0.74	0.50	0.67	0.51	0.51	0.75	0.69	0.45

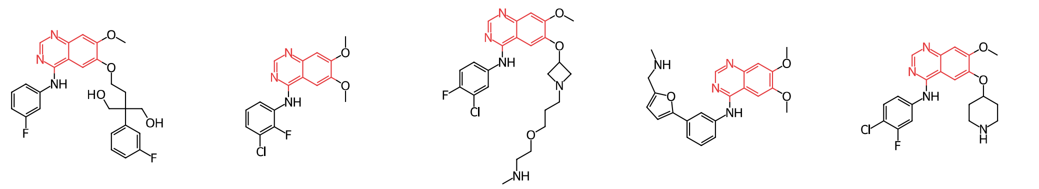

Pred.	7.80	70.1	11.8	7.88	68.1	21.9	7.59	74.6	7.22	7.54	79.3	9.56	7.85	74.3	3.36
Rel.	0.70	0.53	0.53	0.74	0.56	0.63	0.75	0.64	0.48	0.65	0.58	0.58	0.68	0.49	0.49

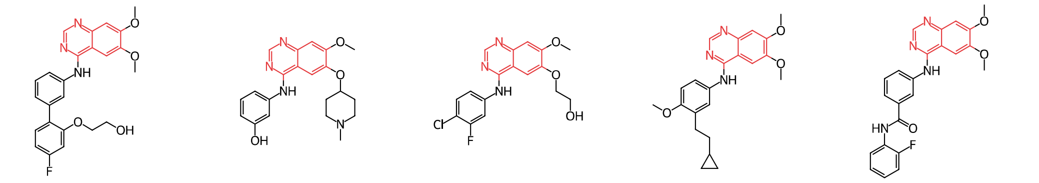

Pred.	7.60	69.4	14.0	7.79	67.4	4.58	7.52	67.4	14.5	7.39	66.5	10.3	7.31	66.8	11.8
Rel.	0.66	0.55	0.55	0.69	0.55	0.55	0.85	0.59	0.51	0.65	0.54	0.54	0.72	0.60	0.60

Inhibitory activity (pIC_{50}) | Metabolic stability (1h remaining (%)) | Permeability ($\mu cm\ s^{-1}$)

Figure S3. Examples of designed molecules with high reward values. Values of predicted properties (Pred.) and prediction reliability, i.e., the maximum Tanimoto similarity with the training data, of each property (Rel.) are shown in each generated molecule. The highlighted areas of molecules represent the quinazoline substructure, a characteristic substructure of known inhibitors for epidermal growth factor receptor (EGFR). Inhibitory activity against EGFR (negative logarithm of the half-maximal inhibitory concentration: pIC_{50}), metabolic stability (remaining percentage in one hour), and membrane permeability ($\mu cm\ s^{-1}$) are colored in blue, green, and red, respectively.

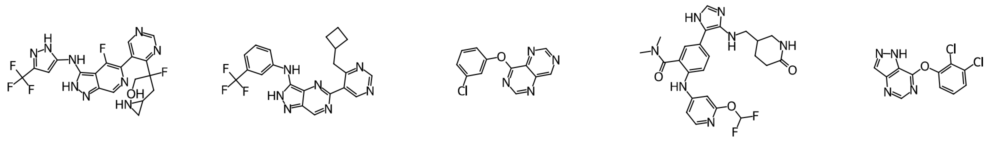

Pred.	7.33	73.3	4.31	7.22	89.8	5.59	7.67	62.0	11.9	7.35	78.8	3.60	7.25	67.4	27.6
Rel.	0.20	0.20	0.21	0.33	0.31	0.28	0.54	0.29	0.50	0.25	0.23	0.25	0.34	0.38	0.38

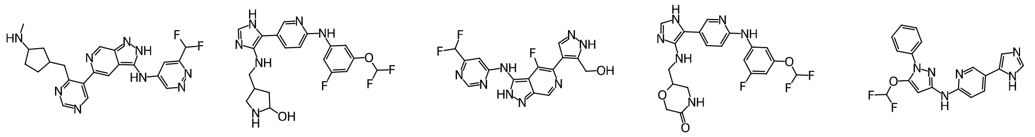

Pred.	7.10	86.5	6.66	7.20	75.6	3.23	7.24	85.9	2.79	7.06	79.8	3.74	6.87	75.2	5.02
Rel.	0.21	0.19	0.21	0.24	0.23	0.26	0.24	0.19	0.20	0.25	0.24	0.25	0.22	0.22	0.21

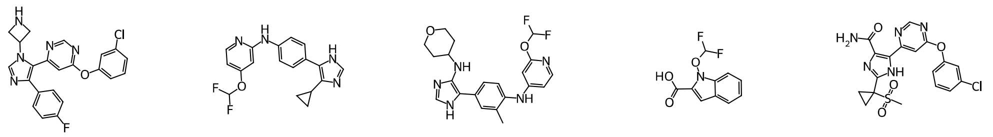

Pred.	6.85	67.2	15.1	6.93	82.4	2.82	6.67	76.1	6.34	6.53	83.2	6.66	6.56	76.2	4.54
Rel.	0.37	0.23	0.33	0.33	0.29	0.24	0.25	0.23	0.29	0.27	0.26	0.33	0.36	0.24	0.29

Inhibitory activity (pIC_{50}) | Metabolic stability (1h remaining (%)) | Permeability (μcm s^{-1})

Figure S4. Examples of molecules designed without reliability consideration. The values under each molecule represent the same ones as those in Fig. S3.

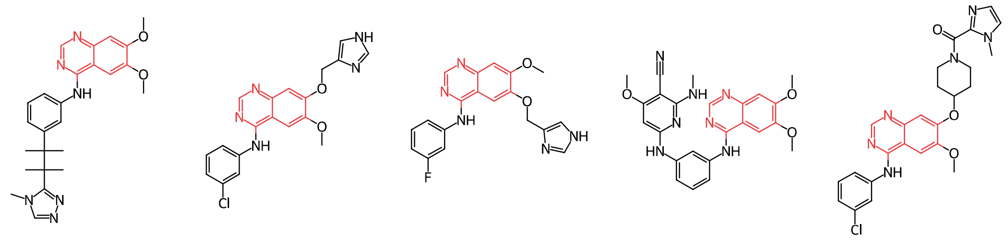

Pred.	8.21	73.6	7.50	8.14	75.9	14.7	8.12	71.6	11.4	8.01	74.1	8.12	7.99	74.2	8.38
Rel.	0.63	0.56	0.56	0.74	0.60	0.60	0.74	0.60	0.60	0.65	0.57	0.57	0.73	0.45	0.50

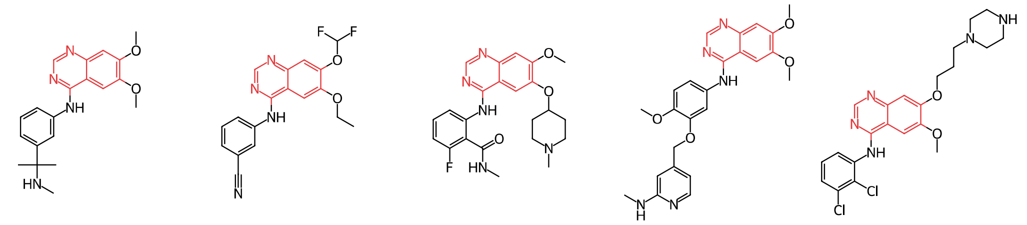

Pred.	7.89	74.9	9.63	7.89	87.0	6.72	7.79	69.6	7.25	7.68	77.7	9.60	7.91	75.7	4.25
Rel.	0.76	0.66	0.66	0.68	0.47	0.47	0.69	0.52	0.63	0.68	0.51	0.51	0.70	0.43	0.50

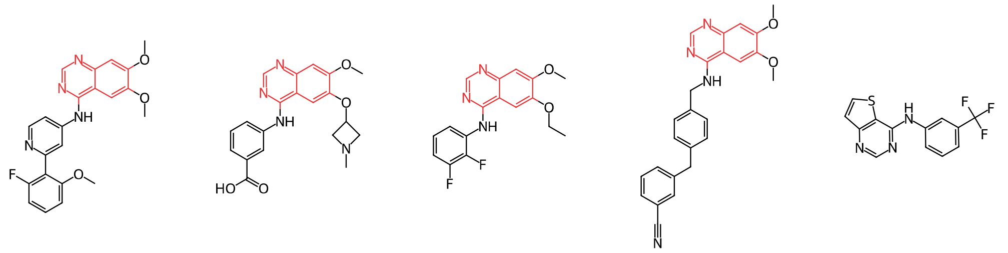

Pred.	7.76	70.0	10.9	7.81	88.7	4.53	7.76	68.5	18.9	7.40	77.1	8.13	7.49	67.9	8.91
Rel.	0.64	0.58	0.58	0.63	0.53	0.53	0.76	0.52	0.52	0.67	0.43	0.43	0.67	0.45	0.45

Inhibitory activity (pIC_{50}) | Metabolic stability (1h remaining (%)) | Permeability ($\mu cm\ s^{-1}$)

Figure S5. Examples of molecules designed by DyRAMO (Dynamic Reliability Adjustment for Multi-objective Optimization) in case removing approved drugs from the training data of property prediction models. The highlighted areas of molecules represent the quinazoline substructure. The values under each molecule represent the same ones as those in Fig. S3.

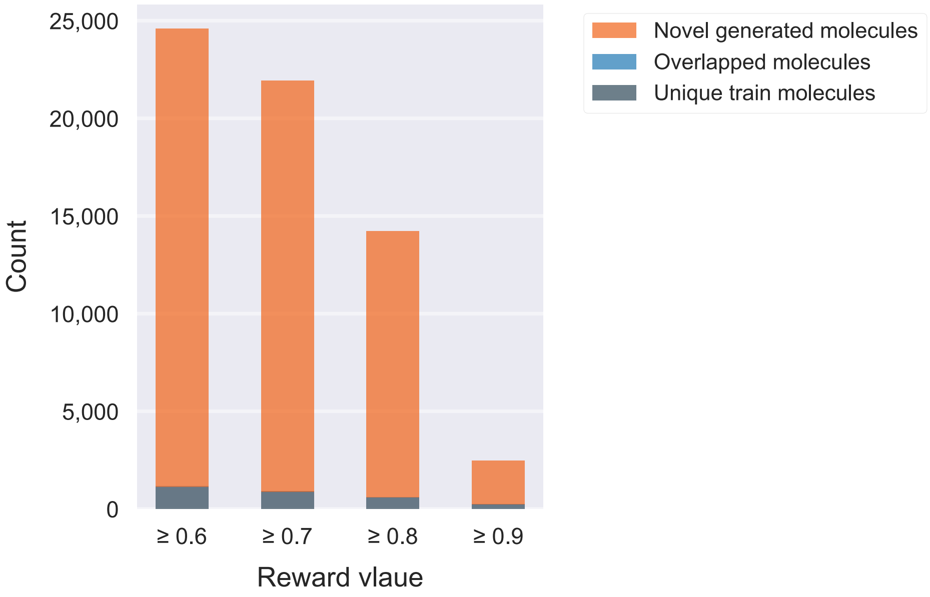

Figure S6. Comparison of molecule counts across reward value thresholds. The generated molecules (in case removing approved drugs from the training data of the prediction models) and training molecules were categorized into three groups: generated molecules that are not included in the training data ("novel generated molecules"), molecules that are found only in the training data (“unique training molecules"), and molecules that overlap between the generated set and the training data (“overlapped molecules”). Bars represent the count of molecules in three categories: novel generated molecules (orange), unique training molecules (dark blue), and overlapped molecules (light blue). Molecule counts are categorized by reward value thresholds (0.6, 0.7, 0.8, and 0.9). Here, the counted generated molecules were selected from the top 10 DSS (Degree of Simultaneous Satisfaction of prediction reliability and multiple property optimizations) score designs across all designs and were filtered to include only those that satisfied the applicability domains (ADs) specific to each exploration step during processes of DyRAMO (Dynamic Reliability Adjustment for Multi-objective Optimization). Similarly, the training set molecules included in the count were those that satisfied one or more of these same ADs, ensuring consistency in the comparison.

Table S1. Molecule counts of the three categories. The table summarizes the number of molecules categorized into novel generated molecules, overlapped molecules, and unique training molecules. These results correspond to the data presented in Figure S6.

Reward value	≥ 0.6	≥ 0.7	≥ 0.8	≥ 0.9
Novel generated molecules	23,477	21,065	13,638	2,230
Overlapped molecules	39	33	20	2
Unique training molecules	1,116	872	587	249

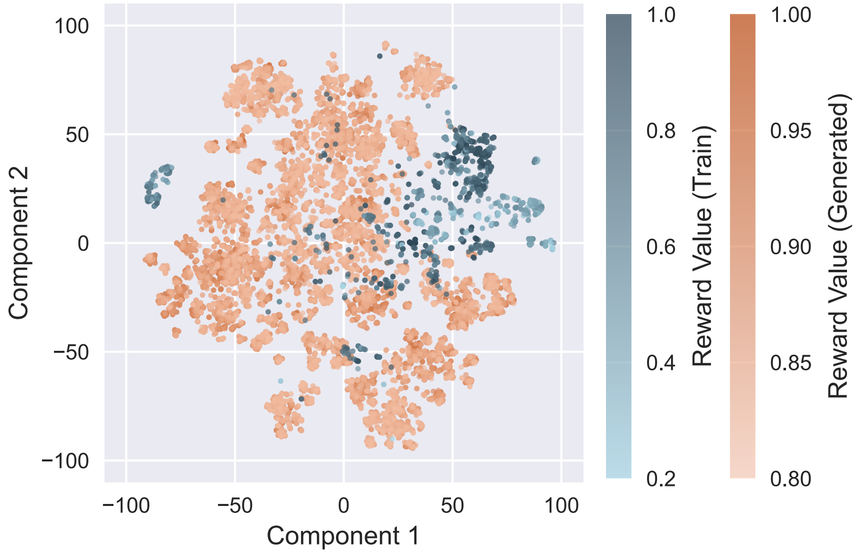

Figure S7. Chemical space distributions for the generated and training molecules in case removing approved drugs from the training data of the prediction models. The plots were generated by applying t-SNE to the distance matrix constructed from Morgan fingerprints and Tanimoto similarity. Orange points represent 10000 molecules with high reward values from novel generated molecules, while blue points represent unique training molecules and overlapped molecules. The color intensity corresponds to reward values, with darker shades indicating higher values.

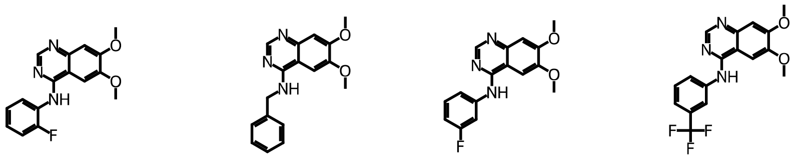

	Exp.: 7.38			Exp.: 8.20			Exp.: 7.79			Exp.: 8.71		
Pred.	6.37	57.7	27.2	6.85	55.6	30.6	7.26	72.9	21.2	7.06	67.1	19.2
Rel.	0.77	0.53	0.46	0.81	0.40	0.35	0.64	0.48	0.41	0.66	0.44	0.43

Figure S8. Examples of molecules reproduced as a result of molecular design when similar molecules across the three datasets are removed. The values under each molecule represent the same ones as those in Fig. S3.

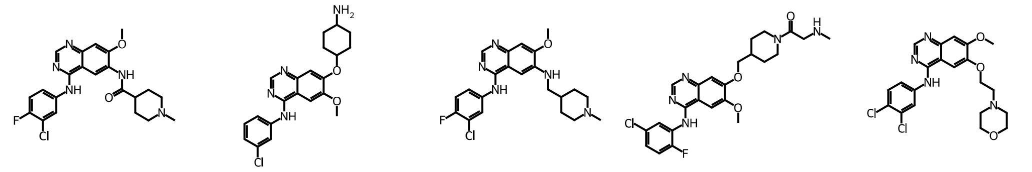

Pred.	8.54	77.6	13.0	8.33	95.1	10.8	7.95	73.8	17.8	7.91	0.76	11.9	7.94	65.8	24.3
Rel.	0.68	0.55	0.46	0.69	0.43	0.48	0.79	0.60	0.42	0.76	0.58	0.50	0.92	0.79	0.40

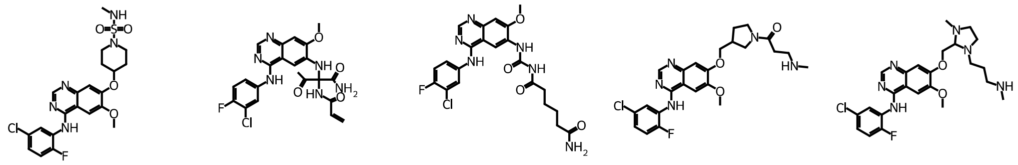

Pred.	8.36	73.9	7.95	8.11	70.8	9.52	8.00	81.8	11.0	7.89	76.0	10.5	7.90	67.9	11.6
Rel.	0.85	0.45	0.55	0.72	0.44	0.37	0.76	0.50	0.40	0.70	0.54	0.46	0.66	0.50	0.42

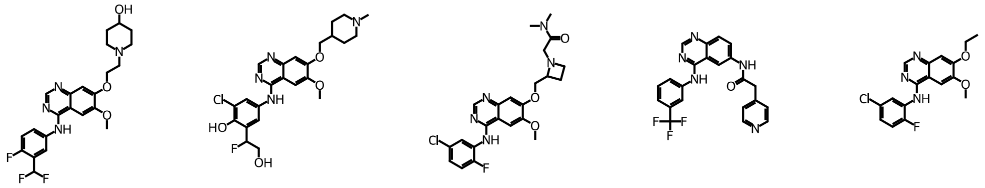

Pred.	7.65	77.6	17.2	7.86	72.2	4.45	7.62	84.5	15.7	7.65	71.8	13.5	7.68	77.7	31.8
Rel.	0.68	0.61	0.43	0.68	0.56	0.40	0.65	0.49	0.47	0.66	0.44	0.44	0.70	0.49	0.43

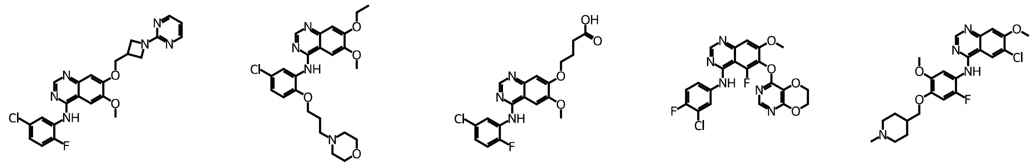

Pred.	7.43	93.6	22.3	7.23	76.1	19.0	7.49	65.4	7.13	7.21	69.5	20.6	7.12	69.6	22.2
Rel.	0.72	0.54	0.43	0.83	0.69	0.41	0.66	0.50	0.44	0.69	0.46	0.29	0.80	0.73	0.44

Inhibitory activity (pIC_{50}) | Metabolic stability (1h remaining (%)) | Permeability (µcm s^{-1})

Figure S9. Example of molecules with high reward values designed when similar molecules across the three datasets are removed. The procedure for selecting the shown molecules, the same as that of Fig. 3c, is as follows. First, molecules that exceeded the set reliability levels were obtained from ten explorations with high DSS (Degree of Simultaneous Satisfaction of prediction reliability and multiple property optimizations) scores. Subsequently, molecules that passed two filters, the rule of five filter and the PubChem filter, were extracted. Finally, k-means clustering (k=20) was performed, and the molecules with the highest reward from each cluster were selected.

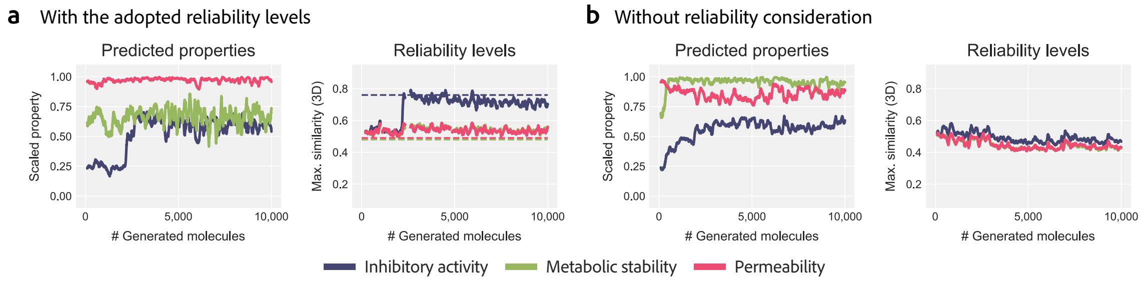

c Designed molecules with high reward values

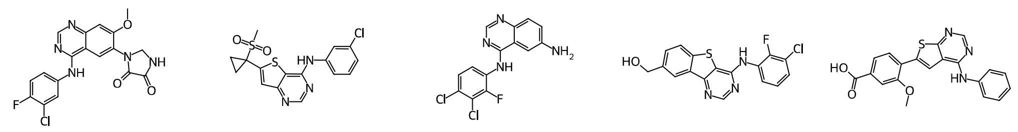

Pred.	8.18	84.2	8.83	8.12	71.3	18.5	7.80	67.2	15.7	7.58	74.9	7.17	7.49	74.8	10.0
Rel. (3D)	0.79	0.71	0.71	0.80	0.62	0.62	0.80	0.63	0.59	0.76	0.58	0.58	0.90	0.52	0.54

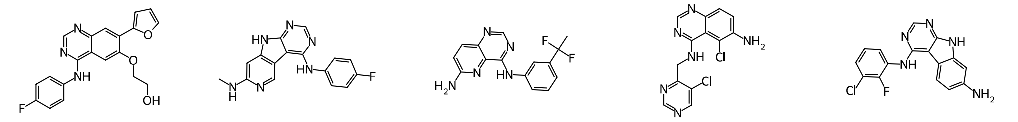

Pred.	7.54	68.8	19.9	7.38	73.1	6.41	7.37	80.4	5.57	7.34	69.1	16.1	7.24	73.4	7.23
Rel. (3D)	0.74	0.64	0.64	0.76	0.56	0.52	0.90	0.66	0.66	0.73	0.59	0.55	0.74	0.53	0.54

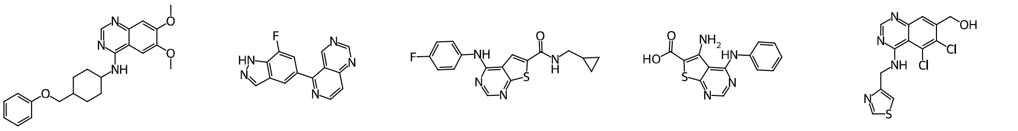

Pred.	7.21	73.4	12.3	7.28	68.3	14.6	7.25	68.5	18.9	7.14	72.3	8.73	7.40	63.2	9.52
Rel. (3D)	0.79	0.54	0.60	0.72	0.60	0.60	0.80	0.60	0.61	0.74	0.64	0.61	0.77	0.52	0.52

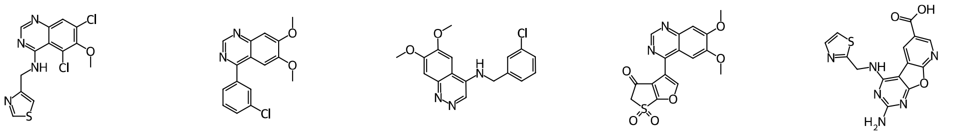

Pred.	7.03	70.3	15.2	7.18	65.9	34.0	6.79	73.3	7.81	6.66	72.8	13.4	6.57	65.4	4.51
Rel. (3D)	0.73	0.57	0.57	0.83	0.68	0.68	0.85	0.63	0.63	0.72	9.58	0.58	0.72	0.53	0.60

Inhibitory activity (pIC_{50}) | Metabolic stability (1h remaining (%)) | Permeability ($\mu cm\ s^{-1}$)

Figure S10. Results of the molecular designs with adjusted reliability levels by DyRAMO (Dynamic Reliability Adjustment for Multi-objective Optimization) (a, c) and without considering prediction reliability (b) in case a 3D-based similarity metric was employed as a criterion for applicability domains (ADs). As a 3D similarity metric, we employed the Tanimoto combo similarity, the sum of shape-based Tanimoto similarity

and pharmacophore-based Tanimoto similarity (commonly referred to as color Tanimoto similarity). To calculate the Tanimoto Combo similarity, we employed shapescreen[2], an open source software for a 3D shape-based similarity scoring. The 3D molecular conformations that are required for this computation were generated using the ETKDGv3 method[3] in RDKit. Apart from the criterion for ADs, conditions for molecular designs and Bayesian optimization remained identical to those described in Section 2.2. **a, b,** The left panels show the moving averages of the predicted properties, with predicted values scaled from 0 to 1. The right panels show the moving averages of prediction reliability, represented as the Tanimoto combo similarity values (ranging from 0 to 2) divided by 2 between the designed molecules and the training data of each property. These moving averages were calculated from each of the 200 designed molecules. The dotted lines indicate the set reliability levels. The right panel of b is identical to that in Figure 3b in the Results section. **c,** Examples of designed molecules, their predicted properties (Pred.), and their prediction reliability (Rel.), represented as the Tanimoto Combo similarity divided by 2 with respect to the training data for each property.

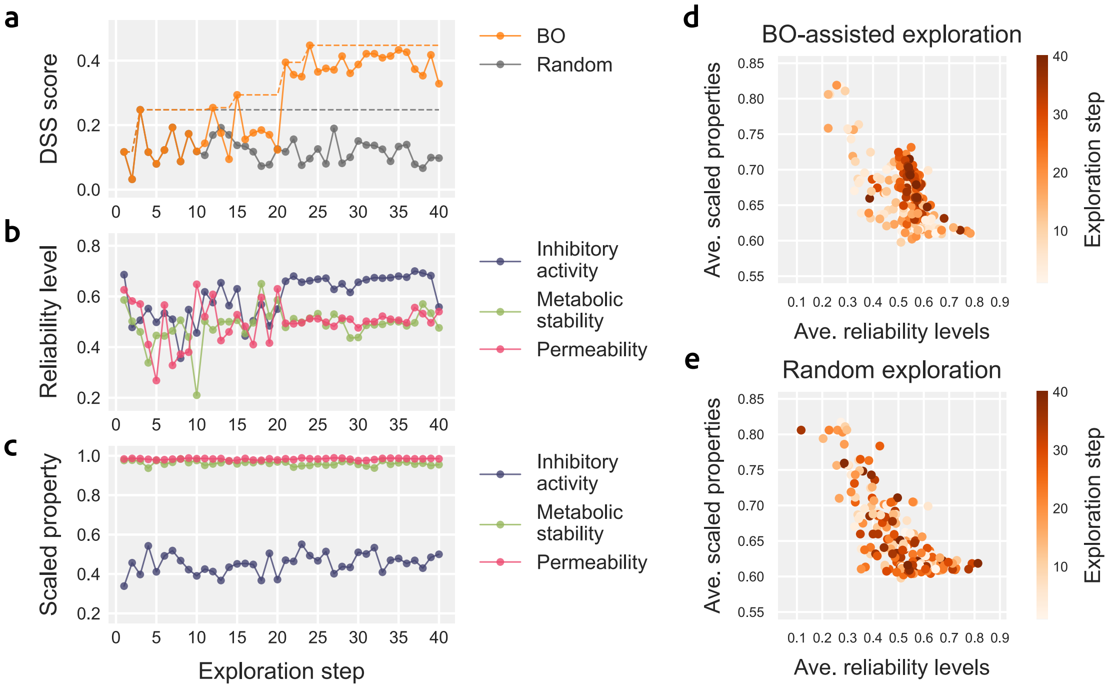

Figure S11. The processes of adjusting reliability levels by Bayesian optimization (BO) and random exploration in DyRAMO (Dynamic Reliability Adjustment for Multi-objective Optimization) in case the Tanimoto combo was employed as a criterion for applicability domains (ADs). **a-c,** a, b, and c show the evolution of the DSS (Degree of Simultaneous Satisfaction of prediction reliability and multiple property optimizations) score, reliability levels, and scaled properties of designed molecules, respectively. **d, e,** The search processes by BO (d) and random exploration (e) in the space of averaged reliability levels and the averaged scaled properties. Each exploration step is color-coded, white at the beginning of the exploration and changing to orange as the exploration progresses.

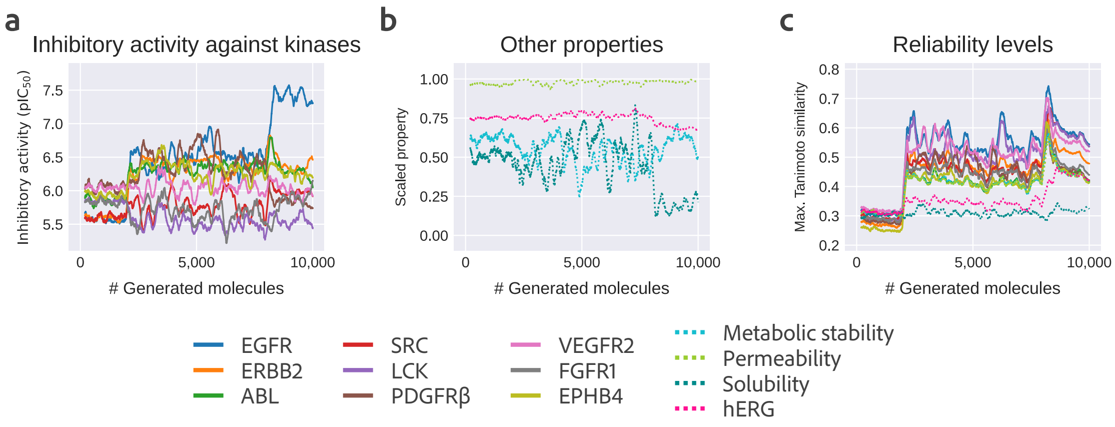

Figure S12. Process of molecular design with the adjusted reliability levels when number of properties to be optimized was 13. **a,** The evolution of inhibitory activity against nine tyrosine kinases. **b,** The evolution of the other four properties that are scaled from zero to one. **c,** The evolution of reliability revels of the 13 properties. As the 13 properties, inhibitory activity against nine proteins from tyrosine kinases and other four properties were selected. For tyrosine kinases, the following nine proteins were selected: epidermal growth factor receptor (EGFR), receptor protein-tyrosine kinase erbB-2 (ERBB2), Abelson tyrosine-protein kinase (ABL), proto-oncogene tyrosine-protein kinase (SRC), lymphocyte-specific tyrosine-protein kinase (LCK), platelet-derived growth factor receptor beta (PDGFRβ), vascular endothelial growth factor receptor 2 (VEGFR2), fibroblast growth factor receptor 1 (FGFR1), and ephrin type-B receptor 4 (EPHB4). Inhibitory activity against EGFR was designed to increase, while inhibitory activities against others were designed to decrease. For the other four properties, metabolic stability, permeability, solubility, and inhibitory activity against human ether-a-go-go-related gene channel (hERG), were selected.

Table S2. The values of adjusted reliability levels when 13 properties are to be optimized.

Property	EGFR	ERBB2	ABL	SRC	LCK	PDGFRβ	VEGFR2
Reliability level	0.5	0.3	0.3	0.3	0.5	0.3	0.3
Property	FGFR1	EPHB4	Stab	Perm	Sol	hERG	
Reliability level	0.4	0.3	0.4	0.4	0.3	0.3	

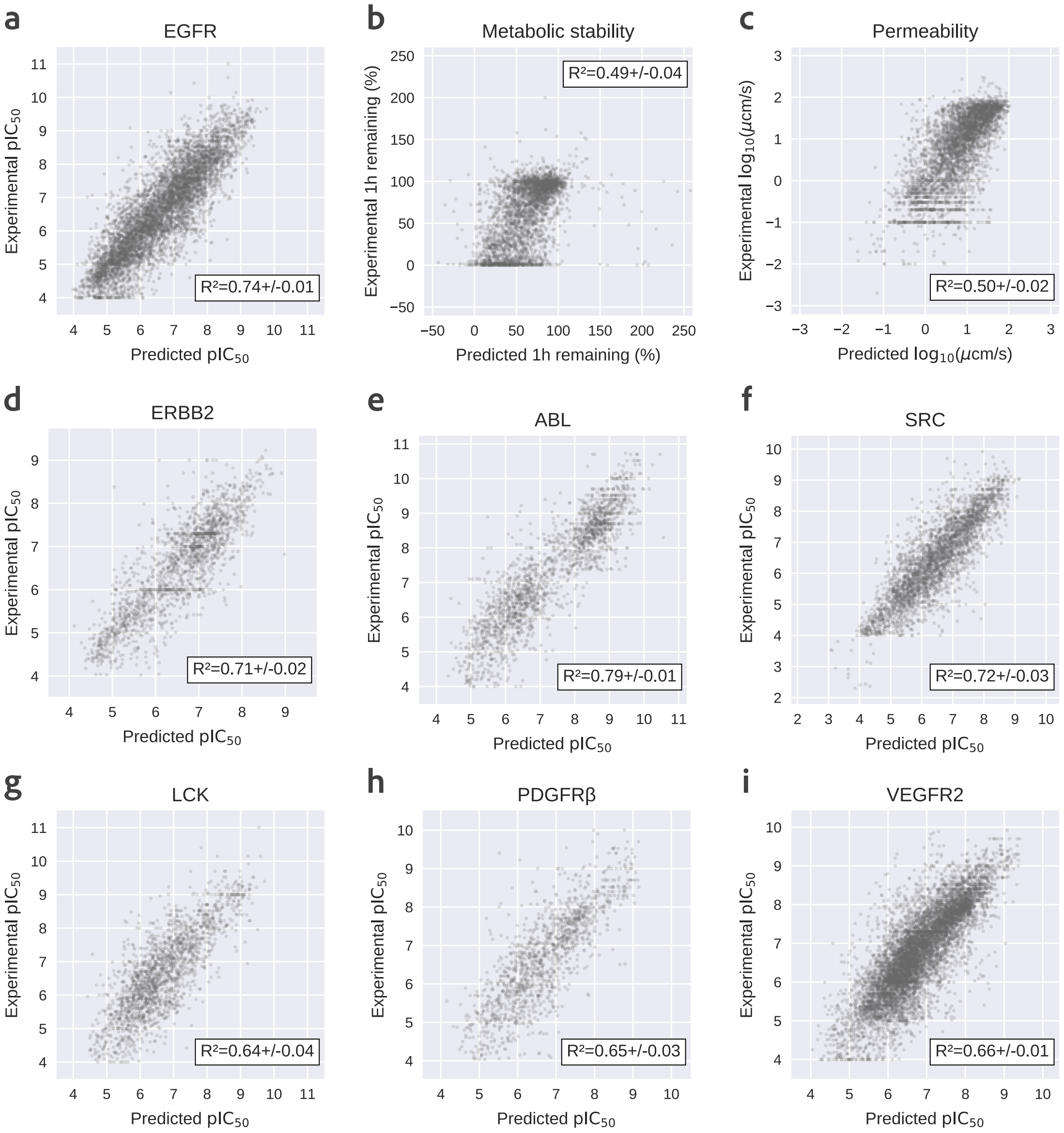
a
EGFR
Experimental pIC50
Predicted pIC50
R²=0.74+/-0.01
b
Metabolic stability
Experimental 1h remaining (%)
Predicted 1h remaining (%)
R²=0.49+/-0.04
c
Permeability
Experimental log10(μcm/s)
Predicted log10(μcm/s)
R²=0.50+/-0.02
d
ERBB2
Experimental pIC50
Predicted pIC50
R²=0.71+/-0.02
e
ABL
Experimental pIC50
Predicted pIC50
R²=0.79+/-0.01
f
SRC
Experimental pIC50
Predicted pIC50
R²=0.72+/-0.03
g
LCK
Experimental pIC50
Predicted pIC50
R²=0.64+/-0.04
h
PDGFRβ
Experimental pIC50
Predicted pIC50
R²=0.65+/-0.03
i
VEGFR2
Experimental pIC50
Predicted pIC50
R²=0.66+/-0.01

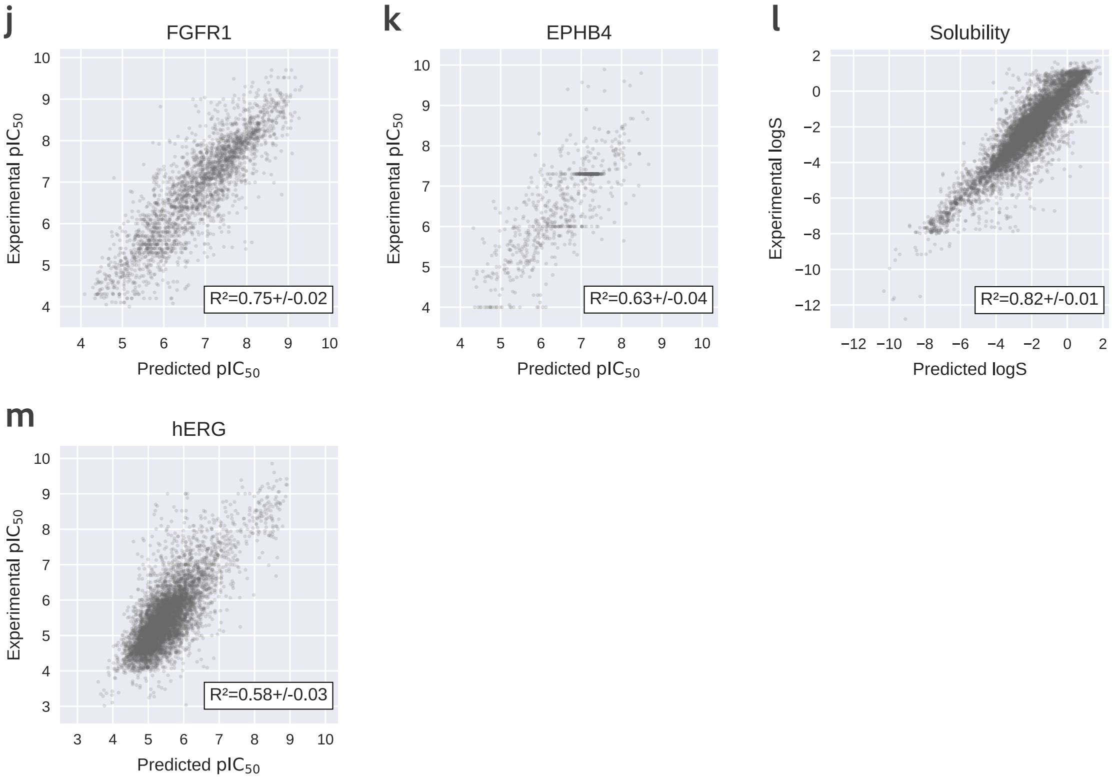

Figure S13 (pages ranging from S15 to S16). Correlation plots of the predicted properties for test data in each fold and corresponding experimental values. a-c represent the results of the properties used when number of properties to optimize was set to three: inhibitory activity against epidermal growth factor receptor (EGFR), metabolic stability, permeability. d-m shows the result of the ten properties added when number of properties to optimize were set to 13: inhibitory activity against receptor protein-tyrosine kinase erbB-2 (ERBB2), Abelson tyrosine-protein kinase (ABL), proto-oncogene tyrosine-protein kinase (SRC), lymphocyte-specific tyrosine-protein kinase (LCK), platelet-derived growth factor receptor beta (PDGFRβ), vascular endothelial growth factor receptor 2 (VEGFR2), fibroblast growth factor receptor 1 (FGFR1), ephrin type-B receptor 4 (EPHB4). solubility, and human ether-a-go-go-related gene channel (hERG) inhibition. The average and standard deviation of the coefficient of determination values over the 5-fold cross validation is shown in the corner of each plot.

References

[1] Brown, N., Fiscato, M., Segler, M. H. & Vaucher, A. C. GuacaMol: benchmarking models for de novo molecular design. *J. Chem. Inf. Model.* **59**, 1096–1108 (2019).

[2] Shapescreen (Chemical Data Processing Toolkit): https://cdpkit.org/v1.1.1/applications/shapescreen.html (accessed December 13, 2024)

[3] Riniker, S. & Landrum, G. A. Better informed distance geometry: using what we know to improve conformation generation. *J. Chem. Inf. Comp. Sci*. **55**, 2562–2574 (2015).
